# A novel technique for fetal heart rate estimation from Doppler ultrasound signal

**DOI:** 10.1186/1475-925X-10-92

**Published:** 2011-10-14

**Authors:** Janusz Jezewski, Dawid Roj, Janusz Wrobel, Krzysztof Horoba

**Affiliations:** 1 Department of Biomedical Signal Processing, Institute of Medical Technology and Equipment, Roosevelta 118, 41-800 Zabrze, Poland

## Abstract

**Background:**

The currently used fetal monitoring instrumentation that is based on Doppler ultrasound technique provides the fetal heart rate (FHR) signal with limited accuracy. It is particularly noticeable as significant decrease of clinically important feature - the variability of FHR signal. The aim of our work was to develop a novel efficient technique for processing of the ultrasound signal, which could estimate the cardiac cycle duration with accuracy comparable to a direct electrocardiography.

**Methods:**

We have proposed a new technique which provides the true beat-to-beat values of the FHR signal through multiple measurement of a given cardiac cycle in the ultrasound signal. The method consists in three steps: the dynamic adjustment of autocorrelation window, the adaptive autocorrelation peak detection and determination of beat-to-beat intervals. The estimated fetal heart rate values and calculated indices describing variability of FHR, were compared to the reference data obtained from the direct fetal electrocardiogram, as well as to another method for FHR estimation.

**Results:**

The results revealed that our method increases the accuracy in comparison to currently used fetal monitoring instrumentation, and thus enables to calculate reliable parameters describing the variability of FHR. Relating these results to the other method for FHR estimation we showed that in our approach a much lower number of measured cardiac cycles was rejected as being invalid.

**Conclusions:**

The proposed method for fetal heart rate determination on a beat-to-beat basis offers a high accuracy of the heart interval measurement enabling reliable quantitative assessment of the FHR variability, at the same time reducing the number of invalid cardiac cycle measurements.

## Background

The main task of fetal monitoring is to ensure that all vital organs are properly supplied with oxygenated blood. Direct measurement of oxygen saturation during pregnancy is not possible, but the risk symptoms can be identified through the fetal heart rhythm analysis. The most accurate measurement of the periodicity in fetal heart activity is limited to the labour, when the acquisition of electrical signals using direct fetal electrocardiography (FECG) is possible [1]. The duration of each cardiac cycle (T_i_) is estimated on the basis of measurement of time interval between successive R-waves in electrocardiogram. As a result the sequence of consecutive interval values is obtained in a form of time event series. Instantaneous fetal heart rate (FHR_i_) values (expressed in beats per minute - bpm) are calculated for each cardiac cycle according to formula: FHR_i _[bpm] = 60000/T_i _[ms].

**Figure 1:**
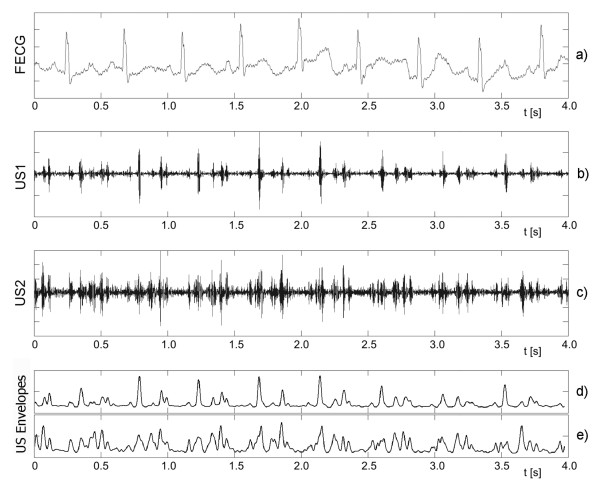
**Fetal heart activity signals**. Four-second segments of the simultaneously acquired signals: a) direct electrocardiogram from an electrode placed on fetal head, b) and c) two Doppler ultrasound signals from two transducers placed separately but focused on the same fetus. Additionally, the envelopes of both Doppler signals are presented as d) and e). Both US signals differ significantly as for the number of cardiac cycle episodes being observed. The periodicity of Doppler signal is much easier to estimate in US1, but only the FECG signal enables explicit recognition of the timing of fetal cardiac events.

The most often used noninvasive acquisition method is the Doppler ultrasound (US) technique [1,2]. It relies on generating the ultrasound beam with frequency of 1 or 2 MHz. The beam, which is formed by US transducer attached to maternal abdomen, penetrates tissues and is reflected from internal body structures wherever it encounters an abrupt change in tissue acoustical impedance. The frequency of the ultrasound wave reflected from moving parts, like valves of the fetal heart, is shifted in accordance with the Doppler effect. The echoes received by US transducer are demodulated to obtain differential frequencies describing the movements within the ultrasound beam. In the demodulated signal the useful components together with various interferences (e.g. caused by fetal movements or maternal blood vessels) are located in the acoustic frequency band. The band-pass filtering of the demodulated signal provides a separation of the useful components (Figure 1b and 1c), and each cardiac cycle is represented by a set of episodes related to particular movements of valves and walls of the fetal heart [3]. The shape of acquired signal and the number of episodes being observed during a single cardiac cycle may vary according to the orientation of the ultrasound beam with respect to the fetal heart (see also the signals representing envelopes in Figure 1d and 1e). Since there are many peaks observed in the signal during each cardiac cycle, the accurate location of the heart beat cannot be explicitly defined and the measurement of beat-to-beat intervals is much more complex than in the FECG signal [4].

In fetal electrocardiogram recorded directly, the shape of QRS complexes does not change significantly from beat to beat, and thus each beat can be easily identified by detecting the evident R-waves [5]. In case of mechanical activity of a fetal heart, both the shape of signal and locations of prominent peaks are varying even between consecutive beats. Hence, in processing of the ultrasound Doppler signal an autocorrelation function (AF) considering a full shape of the analyzed signal is mostly used [6]. In such approach, a graph of similarity between the input signal and its time-shifted version is analyzed. The instantaneous signal periodicity is determined by detection of the AF maximum, whose position indicates the dominant periodicity of signal contained in the window applied. It was confirmed that the AF improves the noise immunity [7,8], although the obtained FHR signal shows a significant decrease of beat-to-beat variability.

The short-term variability describing fluctuations of beat-to-beat intervals is considered to be the most important FHR signal feature indicating appropriate fetal development. The indices describing this variability have been originated from the FECG signal recorded during labour via a direct electrode, and they have been applied without any adaptation to the ultrasound technique. It was shown that the FHR values calculated with the AF are characterized by significant inaccuracy [9], affecting the variability being estimated [10]. It is caused by an averaging nature of the autocorrelation function, which provides a single representative periodicity value but calculated on the basis of all heart beats enclosed in the AF window. The second issue, also affecting the variability measurement, is that autocorrelation technique does not detect events - individual heart beats, which is a standard in FECG signal processing. Therefore, to calculate variability indices consistent with those derived from electrocardiogram, it is necessary to provide the FHR signal in a form of time event series. Such approach was proposed by Tuck [11] and Peters [12]. However, they focused only on accuracy of interval measurement, while from a clinical point of view more important is whether the method improves the reliability of the FHR variability indices.

The aim of our work was to develop a novel processing method for the Doppler ultrasound signal in order to measure periodicity of fetal heart activity with a beat-to-beat accuracy comparable to direct electrocardiography. A special effort was made to reduce the number of incorrectly measured heart intervals, which was achieved by multiple measurement of each cardiac cycle and application of triangular window function for prediction of periodicity in AF. We particularly would like to answer the question if the modern instrumentation together with signal processing technique are able to provide the cardiac cycle measurements with accuracy enough for reliable determination of clinically important signal features. For that purpose, the signals of mechanical heart activity were analyzed using our algorithm for extraction of true time event series representing the consecutive heart beats. The estimated fetal heart rate values were compared to the reference data simultaneously obtained from direct FECG. The final evaluation was based on parameters describing the beat-to-beat variability of the FHR as a signal feature being the most sensitive to any periodicity inaccuracy.

## Methods

**Figure 2:**
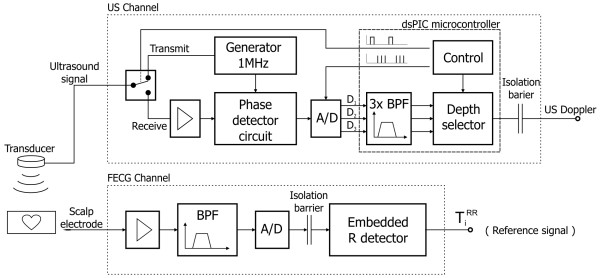
**Instrumentation**. Structure of the developed instrumentation for simultaneous acquisition of both the mechanical and electrical fetal heart activities.

In order to record the signal of mechanical activity of a fetal heart we have developed a prototype acquisition module based on a pulsed ultrasound wave (Figure 2). The advantage of the pulsed wave over the continuous one (where a signal is generated and received at the same time) is the possibility to select the depth of interest in which the movements are detected. The proposed module comprises three main parts: analogue circuit for signal conditioning, A/D converter and digital signal preprocessing module. Analogue part contains the pulsed ultrasound wave generator, control unit and demodulator. The ultrasound transducer is built using 7 piezoelectric crystals of 1 cm in diameter, one positioned centrally and the others in a circle around it. The working frequency is equal to 1 MHz, and the acoustic power of the ultrasound wave does not exceed 1.5 mW/cm^2^.

**Figure 3:**
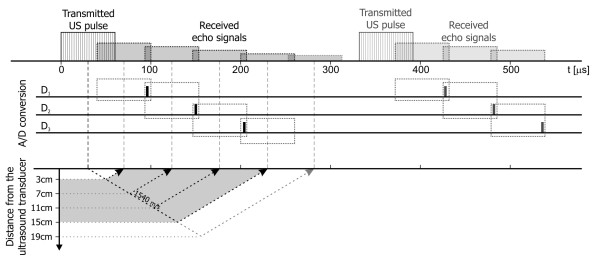
**Ultrasound Doppler signal acquisition technique**. The reflection of acoustic wave on different depths using the pulsed Doppler method. A transmitted pulse of US wave lasting 60 μs penetrates the maternal and fetal body with an average speed of 1540 m/s. The time at which the echo is received depends on a depth of the reflecting object. The received signal is demodulated to obtain the Doppler frequencies, and then A/D converted three times during reception period to cover the depth range of interest - between 3 and 15 cm.

The ultrasound pulse of sixty microsecond width is being sent with repetition frequency of 3 kHz. During the receiving phase the echo signal is acquired by the transducer and converted into an electrical signal, that is next demodulated in a phase detector circuit. The obtained signal is sampled three times during a receiver activity period (which recurs with 3 kHz rate) to cover the depth range of interest, i.e. between 3 and 15 cm. Thus, three digital signals are obtained: D1, D2 and D3 (Figure 3), each of them related to particular depth range. This reduces noise and interferences coming from other organs, and enables using wider US beam facilitating the process of fetal heart beat detection [13].

The signals are preliminary filtered in dsPIC microcontroller to suppress unwanted sources of Doppler signal. Cutting the lower frequencies removes components usually related to fetal movements, whereas the upper limit reduces maternal blood vessel interferences [14]. For this purpose the bandwidth was limited to 100÷600 Hz using FIR filter structure of 120-th order. In the next step, the procedure for depth selection determines the signal (or depth range) in which the fetal heart movements are the most visible. In the obtained Doppler signal sampled with 3 kHz, the mechanical activity of heart is represented as temporary increases of signal amplitude, while its frequency is proportional to a valve/wall movement speed [15]. The signal of the best quality is selected and transmitted via USB interface to personal computer, where processing is carried out using algorithms implemented in Matlab environment.

The determination of signal envelope is crucial for the accuracy of further periodicity measurements, since the most evident peak of AF is seen when the steepness of signal slopes in the envelope is preserved. We calculated the envelope as an amplitude of the analytic signal as defined by (1).

(1) $$A\left(n\right)=|s_{a}\left(n\right)|=\sqrt{s^{2}\left(n\right)+{ŝ}^{2}\left(n\right)}$$

where *s*_*a*_*(n) *is an analytic signal and *ŝ(n) *is a Hilbert transform of *s(n) *signal.

Since the calculation of the envelope is a continuous process, we applied Hilbert transform filter to ensure constant 90° phase shift of the Doppler signal. Finally, the envelope is fed to 120-th order FIR low-pass filter (f_c _= 50 Hz) to remove high frequency components.

Estimation of the FHR signal consists of three steps. The first is responsible for adjustment of window parameters and calculation of the AF, the second determines the instantaneous periodicity of signal by means of improved peak detection algorithm, and the last one calculates the time event series representation of FHR signal using our algorithm for segmentation of instantaneous measurement series.

### Autocorrelation window

The autocorrelation function enables estimating the periodicity of Doppler signal only if at least two corresponding mechanical activity episodes, which belong to two consecutive cardiac cycles, are within the established window [16]. A standard autocorrelation function is defined as follows:

(2) $$\begin{array}{c} R_{n}\left(i\right)=\sum_{j=0}^{N-i}s\left(n+j\right)\cdot s\left(n+j+i\right),\;0\leq i\leq N \end{array}$$

where *s *is the analyzed signal, *n *is the number of first sample in the autocorrelation window and *N *is the AF window length expressed in signal samples. The *R(i) *function expresses the similarity of the analyzed signal and its version shifted in time by *i *samples. As we assumed that the measurable range of fetal heart rate is between 50 and 240 bpm, the periodicity measurement relies on searching the maximal value *R*_*max *_of the function *R(i) *in a range between 250 and 1200 ms.

**Figure 4:**
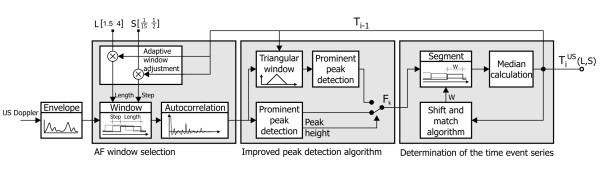
**General scheme of the algorithm for Doppler signal processing**. The final T_i_^US ^signal is a function of two parameters defining the AF: the window length (L) and the shift increment (S), both of them in relation to a cardiac cycle.

In our approach, the window length is updated every time a new cardiac cycle is measured. The length *N *is calculated on the basis of the previous cycle duration and a scaling factor L (N = N(L,T_i_)). During experiments L was being changed between 1.5 and 4 (Figure 4). It is obvious that a long window, which is used to measure low heart rates, requires more computations, but at the same time such long interval does not need to be measured so often. The difference between computational effort needed for short and long intervals can be compensated by higher shift increment for long cycles and lower for short ones. Therefore, our window shift increment is dynamically adjusted as a product of the last cycle duration and scaling factor S (n = n(S,T_i_)). The optimal value of S was searched in a range from 1/15 to 1/2, which means that an average number of measurements per interval was between 2 and 15. Application of adaptive window reduced the effect of periodicity averaging as well as computational complexity of the algorithm [17], whereas repeated AF calculation during a given cardiac cycle improved the noise immunity, and as a consequence the accuracy of measurement [18].

### Peak detection

**Figure 5:**
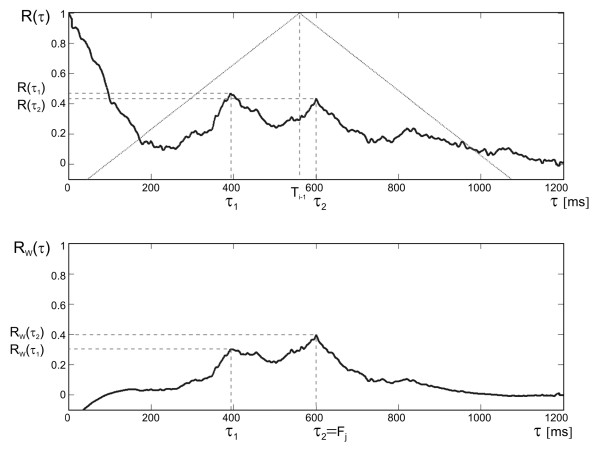
**Improved peak detection algorithm**. General idea of correction of the autocorrelation function using a triangular window. The upper plot presents the autocorrelation R(τ) and the triangular window (W = 1.5) centered according to the last correctly measured interval. The plot below presents the R_W_(τ) function obtained after correction of R(τ). The correction enables the selection of an appropriate interval value τ_2 _even when a false maximum R(τ_1_) was caused by interferences.

If the quality of signal is high enough, the position of AF maximum in current window directly indicates the signal periodicity. Lower peak of AF indicates a lower signal quality (shape similarity), and thus the higher probability of erroneous measurement of cardiac cycle duration [19]. These errors are usually the result of false maxima recognition, caused by the artifacts in the Doppler signal often coming from maternal blood vessels. To prevent incorrect measurements in case of low quality of signal, the prediction of cardiac cycle duration is proposed. When the signal quality decreases, i.e. the height of peak of AF goes below the empirically established threshold (R_max _= 0.6), the AF correction is applied. To this end we used triangular window function with the center in τ = T_i-1_, considered as the expected value of the next cardiac cycle (Figure 5). It means that for arguments close to previously observed cycle duration the height of AF peaks is only slightly changed, while the other peaks are suppressed. Finally, the position of maximum in windowed AF (R_W_) is chosen as the value of instantaneous periodicity F_k_:

(3) $${\text{F}}_{\text{k}}={\text{τ}}_{\text{n}}\left\{{\text{τ}}_{\text{n}}:{\text{R}}_{\text{W}}\left({\text{τ}}_{\text{n}}\right)=\text{max}\left({\text{R}}_{\text{W}}\left(\text{τ}\right)\right)\right\},\;R_{W}\left(\tau \right)=R\left(\tau \right)\cdot \left(1-W\cdot \left|\frac{\tau -T_{i-1}}{T_{i-1}}\right|\right),$$

where *W *stands for the parameter controlling triangular window shape, which was being changed between 0 (no windowing) and 6 (the narrowest window). Any instantaneous periodicity values F_k _for which the peak is lower than the minimal acceptable peak P_TH _= 0.15 are rejected.

### Time event series

The autocorrelation function enables estimation of cardiac cycle duration within a given time window. However, no information on the exact position of heart beats in time is given. Thus, using the AF we obtain a series of values, where each cardiac cycle is represented by a set of periodicity measurements F_k_. This representation has to be converted into time event series using an additional algorithm for segmentation of measurement series F_k_. Then, on the basis of F_k _values contained in each segment, a true value of T_i _which represents successive cardiac cycles is calculated [20].

**Figure 6:**
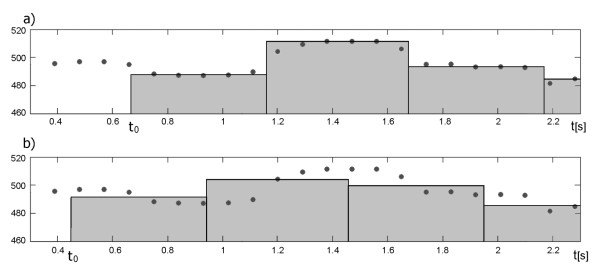
**The influence of segmentation procedure on the beat-to-beat variability**. Example showing a random selection of a starting point for our segmentation algorithm. Two situations are depicted: a) correct match between segments and instantaneous periodicity measurements, b) phase-shifted segmentation causing significant false decrease of beat-to-beat variability in the FHR signal.

In this paper we propose a segmentation algorithm which is based solely on the values of measurement series F_k_. The location of successive segments is continuously adjusted in relation to the measurement series in order to obtain the lowest dispersion of measurements enclosed (Figure 6). The dispersion is calculated as the mean difference between the measurements within the segment and its representative value. The general idea of the segmentation method is based on calculation of the value of *i-*th interval (T_i_) as a median of measurements within a segment of length equal to the previously determined interval duration - from τ_i _to τ_i_+T_i-1_. In the resulting time event series each interval is described by a precisely measured duration T_i _as well as a marker of its occurrence in time. Such T_i _value determines the time marker of the beginning of next segment (τ_i+1_). However, the results depend strictly on the starting point selected, affecting the phase shift of segments in relation to periodicity measurements - F_k _(Figure 6). As a solution, we propose the phase compensation algorithm being independent from random choice of the starting point (the beginning of the first cardiac cycle) and immune to measurement errors which could lead to erroneous location of segments. Each time a new interval value is determined the 'shift and matching' procedure is performed. Thus, the time markers of the three latest segments are shifted and then a mean absolute difference is calculated between the interval values assigned to those segments and the instantaneous periodicity values, which are within their limits. In case when the smallest difference is obtained for non-zero shift value, the appropriate phase correction should be applied to the time marker of beginning of the next interval, whereas the T_i _value remains unchanged.

In details the algorithm for segments shift and matching is as follows:

Starting conditions: *i = 1*, beginning of the first interval *τ*_*1 *_= *0 *and *T*_*0 *_= *F*_*1 *_(zero interval equal to the first periodicity measurement).

**Figure 7:**
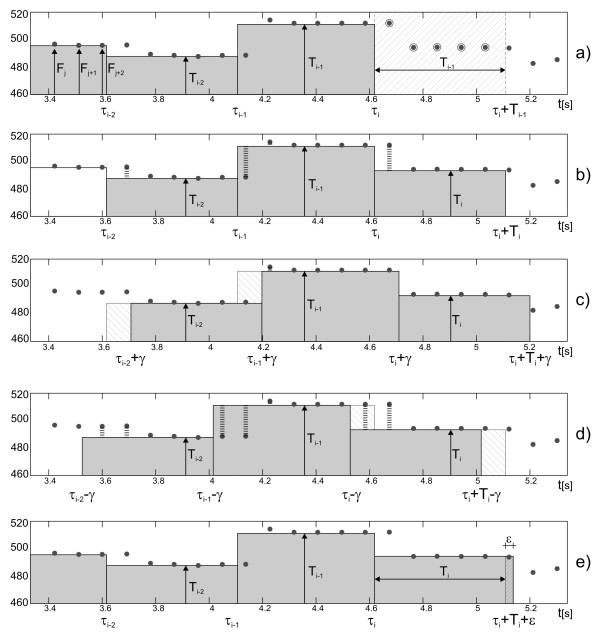
**Graphic illustration of the segments shift and matching method**. The first picture a) presents the way in which a new cardiac cycle duration is calculated, next three b), c), d), depict the principle of correction factor determination. If the mean difference between values F_k _and the corresponding values of T_i _intervals decreases after the interval displacement by either γ or -γ (where γ is equal to temporary window shift increment), the correction factor should be applied into the segment duration. In that case, the last interval duration is corrected by a factor ε = γ/4 or ε = -γ/4 respectively, which can be seen in e).

Step 1 Calculate interval value T_i _as the median value from measurements made between τ_i _and τ_i_+T_i-1 _(Figure 7a).

Step 2 Calculate the mean difference between values of instantaneous periodicity F_k _measured within segment from τ_i-2 _to τ_i_+T_i _and the values T_i_, T_i-1_, T_i-2 _corresponding to segments: τ_i-2_÷τ_i-1_, τ_i-1_÷τ_i_, τ_i_÷τ_i_+T_i _respectively (Figure 7b).

Step 3 Repeat the same calculations for cardiac cycle markers τ_i-2_, τ_i-1_, τ_i _shifted in time by *γ = ±Shift*, where *Shift *stands for temporary shift increment, defining time interval between periodicity measurements F_k _(Figure 7c and 7d).

Step 4 If the least mean difference is obtained in case of markers displacement, set the correction factor ε to 1/4·γ, otherwise to 0 (Figure 7e).

Step 5 Calculate the time of the next interval beginning τ_i+1 _= τ_i _+ T_i _+ ε.

Step 6 Update *i = i+*1 and repeat the calculations for subsequent intervals (return to 1).

Steps 2÷4 are skipped during calculation of first two intervals.

### Interval validation

To reduce large measurement errors caused by a signal interferences which were not removed by the AF (i.e. related to erroneous heart rate doubling or the temporary transition to maternal heart rate), an additional validation process for rejecting of the suspicious T_i _intervals has to be applied [11,21]. As the commonly used validation criteria [21] accept too wide range of heart rate changes, we applied more precise rules for verification of the interval values [22]. The T_i _values should fulfill the condition:

(4) $$T_{i-1}-0.10\cdot {\text{Δ}}_{i-1}<T_{i}<T_{i-1}+0.15\cdot {\text{Δ}}_{i-1}$$

where

$${\text{Δ}}_{\text{i}-1}=\left\{\begin{array}{cc} {\text{T}}_{\text{i}-1}-300\;\text{ms} & \text{for}\\ 20\;\text{ms} & \text{for} \end{array}\begin{array}{c} \;{\text{T}}_{\text{i}-1}\geq 320\;\text{ms}\\ \;{\text{T}}_{\text{i}-1}<320\;\text{ms} \end{array}\right)$$

The T_i _is accepted if it belonged to the group of three consecutive intervals fulfilling the (4). The validation is carried out bidirectionally, which means that in the next step intervals are accepted according to their successors. A given interval is considered as incorrect only if it does not meet the criteria in both directions.

## Results

The research material was collected from three patients during labours, using our developed instrumentation. All patients gave their informed consent prior to a participation in the study. Each of three recordings consist of two simultaneously acquired signals: the Doppler US signal from the abdominal transducer and the direct fetal electrocardiogram from electrode placed on fetal head. The total recording time was 82 minutes, although some fragments in which either fetal electrode was disconnected or the ultrasound transducer lost the heart signal, were marked as signal loss and removed. Finally, 68 minutes of recording (with a total number of 8945 intervals detected in FECG) had a quality satisfactory for further analysis (Table 1).

**Table 1:** Reference data statistics

Recording	Duration [min:s]	Signal loss [%]	Number of heart beats	Mean FHR [bpm]	STI Mean ± SD
1	45:26	3.7	6015	132.2	9.17 ± 2.40
2	13:22	0.8	1188	127.7	7.37 ± 0.75
3	9:20	2.1	1742	130.4	6.54 ± 0.65

Descriptive statistics of the reference FHR signals derived from direct fetal electrocardiographic recordings

### Comparison with reference signal

In order to measure the reference T_i_^RR ^intervals in the direct fetal electrocardiogram (sampled at 2 kHz) we implemented a method based on the cross-correlation function. This function was used to find QRS complexes in electrocardiogram by comparing the signal with a template. As the template we used an averaged QRS complex calculated for every one-minute segment of FECG signal. Both the reference and the ultrasound signals were represented in a form of time event series. To assure reliable evaluation of ΔT_i _error, resulting from the ultrasound method, the comparison had to be carried out with the corresponding heart intervals in the reference signal. The synchronization of simultaneously recorded signals was achieved through visual adjustment of the reference signal in order to obtain the best waveforms fit. The measurement error ΔT_i _for a given heart interval was defined as the difference between the value of T_k_^US ^from ultrasound and corresponding reference interval T_i_^RR ^(according to the middle point of the reference interval duration):

(5) $$\begin{array}{cc} \text{Δ}T_{i}={T_{k}}^{US}-{T_{i}}^{RR}, & \text{for }k\text{ meeting condition }{\tau_{k}}^{US}\leq 0.5\cdot \left({\tau_{i}}^{RR}+{\tau_{i+1}}^{RR}\right)<{\tau_{k+1}}^{US} \end{array}$$

As the research material consisted of three recordings, the results were describing the cumulative data (a set of ΔT_i _values obtained from all three recordings).

To quantify the short-term variability of the FHR signal we applied the STI index proposed by de Haan [23], which was chosen from many others as being the most reliable measure of the beat-to-beat changes [24]. The STI index was defined as follows:

(6) $$STI=IQR\left(\varphi_{i}\right),$$

where *φ*_i _= *arctg*(*T*_*i*_/*T*_*i*__-1_), *IQR *- interquartile range.

The index was calculated within separate one-minute signal windows. Since its value changes considerably between subsequent windows, the relative error δSTI was more suitable:

(7) $$\delta \;STI_{j}=\left(\;ST{I_{j}}^{US}-ST{I_{j}}^{ECG}\right)\;∕ST{I_{j}}^{ECG},$$

where *j *indicates the j-th minute of a record being analyzed.

### Triangular window adjustment

**Figure 8:**
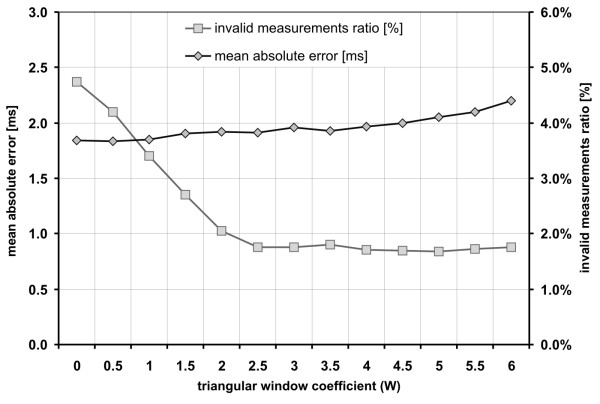
**Triangular window width**. The chart presents the influence of triangular window parameter *W *on the mean absolute interval error and on the number of invalid measurements rejected during the validation stage. The T^US ^signal was calculated for *W *values varying from 0 (no windowing) to 6 (the most narrow window), while the AF window length L = 2.5 and shift increment S = 1/5.

To select an appropriate value of the triangular window coefficient *W*, we investigated its influence on the interval measurements error as well as the number of rejected invalid measurements (Figure 8). The invalid measurements ratio was defined as a percentage of the total time of episodes with invalid intervals in relation to the time of recording. The duration of such episode was measured from the position of invalid interval marker to the next correct heart interval onset.

A significant decrease of invalid measurements ratio (from 4.7 to 1.7%) was observed for *W *values being changed from 0 to 2.5. However, at the same time the interval measurement error slightly increased (from 1.83 to 1.91 ms). It can be explained by the fact that the fragments of signal, in which the correct measurement was only possible with application of the triangular window, usually contained strong interferences.

Further increase of *W *values (from 3 to 6) did not affect the invalid measurements ratio, while the interval error ΔT_i _increased up to 2.20 ms, because the window was too narrow to follow the beat-to-beat changes of FHR. Finally the *W *= 2.5 as an optimal value was established for further signal processing.

### T_i _measurement accuracy

**Figure 9:**
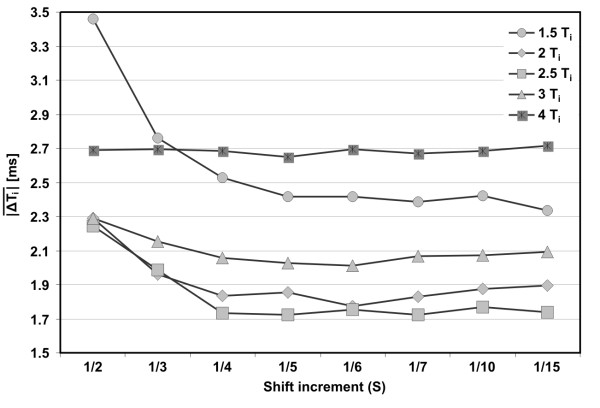
**Shift increment selection**. The mean absolute error of interval measurement $\bar{|\text{Δ}{\text{T}}_{\text{i}}|}$ as a function of window shift increment S (from 1/2 to 1/15) obtained for five different lengths of AF window (1.5, 2, 2.5, 3 and 4 T_i_).

The influence of shift increment on the accuracy of T^US ^event series extraction was analyzed by means of absolute interval measurement error $\bar{\;|\text{Δ}T_{i}|\;}$ and the results are presented in Figure 9. The lowest error values were obtained for 4 or 5 measurements per interval, while for less than 4 measurements (S > ¼) a considerable increase of $\bar{\;|\text{Δ}T_{i}|\;}$ was observed. Thus, we can assume that four is the minimal number of measurements per interval for which the algorithm still works properly. Since an increase of measurement rate above 5 per interval does not improve the accuracy, this number was established for further experiments (S = 1/5).

**Figure 10:**
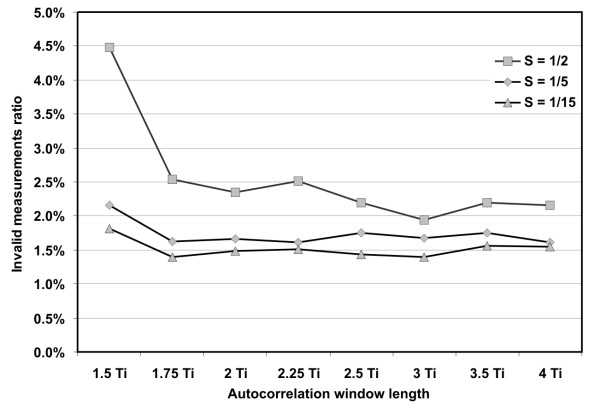
**Invalid measurements ratio**. The relation between the AF window length and the invalid measurements ratio in the analyzed recordings. The results were obtained for S = 1/5, assumed to be the optimal shift increment, as well as for two boundary values in the range investigated.

We have investigated the immunity of our algorithm to interferences and low signal quality, analyzing the relation between the AF window length and the invalid measurements ratio (Figure 10). The results obtained for the established value of shift increment (S = 1/5) showed that the window length of 1.5 T_i _was too short for reliable estimation of signal periodicity, since it led to the highest number of invalid measurements. For windows longer than 2 T_i _the ratio remained below the level of 1.7%, but no visible tendency could be noticed together with the increasing window length. Thus, considering the number of invalid measurements, there was no reason to extend the window above the length of 2 T_i_.

Using the shift increment S = 1/5 we investigated the relation between interval error and the window length *L*. We noticed that for different *L *not only the mean absolute error is changing but also the distribution of error values, the results are presented in Table 2. The window length set in range from 1.75 to 2.5 T_i _results in similar error values. The shortest one (1.5 T_i_) still enables us to calculate at least 50% of intervals with accuracy better than 1.1 ms. However, the number of erroneously measured intervals is considerably increased (the SD value about two times higher). Whereas, in case of windows longer than 2.5 T_i_, the measurement error increases monotonically regarding both the median value and the standard deviation.

**Table 2:** The accuracy of interval measurement

Window length	$\bar{\text{Δ}}T_{i}\pm \text{SD}$**[ms]**	$\bar{|\text{Δ}T_{i}}|$**[ms]**	Median (|ΔT_i_|) [ms]
1.5	1.48 ± 6.82	2.58	1.04
1.75	0.24 ± 3.35	1.73	1.00
2	0.18 ± 3.05	1.68	1.02
2.25	0.08 ± 3.51	1.82	1.07
2.5	0.09 ± 3.59	1.94	1.15
3	0.19 ± 3.67	2.11	1.30
3.5	0.33 ± 4.13	2.40	1.45
4	0.48 ± 4.54	2.70	1.60

The descriptive statistics of differences between intervals derived from the Doppler ultrasound signal and the reference direct fetal electrocardiogram. The results were obtained on the basis of 8945 interval measurements.

### STI determination accuracy

**Figure 11:**
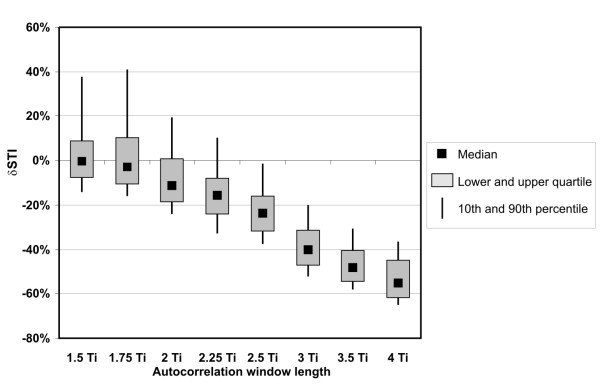
**Short-term variability error**. Relationship between the autocorrelation window length and the relative error of FHR variability index calculation δSTI. The error distribution is presented as a median value, lower and upper quartiles, together with 10th and 90th percentile as a measure of dispersion.

To evaluate the accuracy of short-term FHR variability assessment, we investigated the relation between the STI index and the AF window length (Figure 11). We noted that application of longer window causes significant decrease of short-term variability in FHR signal. In the worst case (for window 4T_i_) the value of median error dropped below -50% making STI indices useless as leading to erroneous clinical diagnosis. On the other hand, the plus sign of the error value indicates the overestimation of FHR variability, which is connected with a high risk of false assessment of fetal wellbeing. Concluding, the optimal window length of 2 T_i _caused the bias error on -10% level, and at the same time minimizes the risk of missing fetal distress signs.

### Comparison with others

In order to evaluate our approach, we compared it with the method of beat-to-beat FHR determination proposed by Peters et al. [12], as the only one providing the time event series representation and being described in details, which enable its implementation. As opposed to ours, it is based on single measurement of a given cardiac cycle, by means of preliminary detection of heart beat locations in time, for which a signal window is set. Then, an autocorrelation procedure is applied to each of these windows, providing one instantaneous periodicity measurement for each detected heart beat. Implementation of this method, supported by the same validation rules as applied to our method (4), enabled objective comparison of the accuracy of cardiac interval measurement, the STI indices calculation as well as the number of rejected invalid measurements.

In [12] the method was evaluated basing on a short recording (comprising only 267 cardiac cycles), revealing a high consistency of intervals simultaneously derived from the ultrasound method and the direct electrocardiography (correlation coefficient r = 0.977). When we implemented this method for processing of our recordings (comprising 8945 cardiac cycles) the results were better (r = 0.992, Table 3). Applying the window length of two cardiac cycles, our method provided the same correlation coefficient. Also the mean absolute error of interval measurement obtained using our research material was equal for both methods (1.91 ms). The most noticeable difference between the methods concerns the number of invalid measurements, which was as low as 1.6% in our approach, whereas for Peters' method it was 5.4%. Because the difference in the number of rejected intervals between the two methods was so high (about three times higher for the implemented single measurement method), the comparison was repeated for normalized number of rejected intervals. We removed all these intervals which were marked as invalid for at least one method. This time the interval measurement accuracy for our method (mean absolute error of 1.73 ms) was higher than accuracy of the single measurement method (1.89 ms).

**Table 3:** Comparison with other method

Method:	Proposed	**Peters **[12]
Invalid measurement ratio [%]	**1.6**	5.4
$\bar{|\text{Δ}{\text{T}}_{\text{i}}|}\;\;\;\left[\;\text{ms}\right]$ (Original signals)	**1.91**	**1.91**
$\bar{|\text{Δ}{\text{T}}_{\text{i}}|}\;\;\;\left[\text{ms}\right]$ (Normalized signals)	**1.73**	1.89
Mean(δSTI) [%]	-6.9	**-5.1**
Correl. coeff. r	**0.992**	**0.992**

The parameters describing accuracy of FHR signals determined using the method presented in this paper and the one from Peters et al. Both methods were compared using the research material comprising 8945 intervals.

An alternative approach was presented by Lee et al. [18], who used a sliding window to obtain multiple estimates for FHR corresponding to each cardiac cycle which, comparing with Peters' method, improved the immunity to noise. However, the resulting FHR values were in form of evenly distributed samples. Thus, as we would like to evaluate the accuracy on a beat-to-beat basis (which is required to calculate variability indices), we could not relate our results to this method.

## Discussion

The investigation of the proposed signal processing technique was carried out for lengths of AF window changing from 1.5 to 4 T_i_. For windows shorter than 2 T_i _the correct periodicity measurement is possible only if the window includes two corresponding events of the subsequent cardiac cycles. Fortunately, in the Doppler ultrasound signal each cardiac cycle is represented by a number of different events. Thus, even when some of them are missing, the AF peak remains visible. That is why the results for window length of 1.75 T_i _do not differ too much from those for 2 T_i _window. On the other hand, extending the window from 2 T_i _to even 2.25 T_i _noticeably increases the negative variability error δSTI. We showed that even relatively small errors of interval measurement, caused by AF period averaging, lead to a significant decrease of the FHR variability index STI.

Comparing our method with the one proposed by Peters et al. we noted very similar accuracy of measured intervals. But slightly better results concerning the variability indices were noted for single measurement method (mean relative error of -5.1%). It confirms that reliable evaluation of FHR signals requires also an assessment of short-term variability, since it does not depend directly on the accuracy of interval measurement. Nevertheless, in case of both methods the mean relative error of short-term variability did not exceed -7%, which could be an acceptable result.

In the work [24], the accuracy of the fetal heart rate estimation was evaluated for MT-430 fetal monitor (Toitu, Japan) with built-in autocorrelation function. The authors concluded that even the new-generation fetal monitors are not capable of providing the FHR signals with accuracy enough for reliable beat-to-beat variability assessment. Too long AF window causes considerable averaging of measurements, and in consequence a loss of information concerning the true variability. For that fetal monitor the mean absolute error of intervals was equal to 2.98 ms with standard deviation of 4.18 ms. The relative error, while computing the STI variability index, was equal to -39.5%. These results are similar to those obtained in this work but assuming the window length in a range from 3.5 to 4 T_i_. Nevertheless, our new processing technique significantly improves the accuracy of the variability index calculation if shorter windows are applied. The values of mean absolute error and its standard deviation (obtained for selected window length of 2 T_i_) equal to 1.91 ms and 3.48 ms respectively, are much better than those reported for MT-430 fetal monitor. In consequence, the relative error of the STI calculation was also significantly lowered (-6.9%).

## Conclusions

The results show that autocorrelation technique allows us to obtain reliable parameters describing the beat-to-beat FHR variability, but only when the length of the applied window stays close to two periods of a heart activity. Thus, to achieve the highest accuracy of interval measurement, a precise adaptive algorithm for adjustment of window length is necessary. We noticed that even a shorter window enables to correctly determine the signal periodicity. However, lower accuracy of interval measurement and higher number of intervals rejected by validation criteria make such short window useless. The comparison with another known approach to the FHR estimation [12] revealed that our method offers the same accuracy of interval measurement and slightly higher values of the short-term variability error (slightly higher for our method). However, the biggest advantage of the proposed solution over the other is the three times lower number of invalid measurements.

In a future we are going to record much more Doppler ultrasound signals, especially during pregnancy, where the reference signal will be provided by an indirect electrocardiography, i.e. from electrodes placed on maternal abdomen. Since abdominal signals are much more comfortable for the patients, considerably more recordings should be collected. Therefore, we will be able to test our processing methods with more representative material, as well as to investigate its efficiency during standardized clinical patterns of the Doppler ultrasound signal (e.g. acceleration/deceleration episodes). Another field of interest is to assess the noise immunity of our method, which is a key issue for accurate variability measurement, due to a short autocorrelation windows applied. This research work should help us to develop the optimal signal processing techniques, that could be implemented in a prototype mobile Doppler ultrasound signal recorder developed as an add-on PC card.

## Competing interests

The authors declare that they have no competing interests.

## Authors' contributions

JJ participated in hardware design and carried out the experiments. DR was responsible for the algorithm of periodicity measurement in ultrasound Doppler signal. JW developed phase compensation algorithm. KH was responsible for quantitative analysis of the FHR. All authors contributed in general concept and writing of the paper. All authors read and approved the final manuscript.
